# Clinical outcomes and complications of cementless reverse total shoulder arthroplasty during the early learning curve period

**DOI:** 10.1186/s13018-019-1077-1

**Published:** 2019-02-18

**Authors:** Sungwook Choi, Jong-Hwan Bae, Yong Suk Kwon, Hyunseong Kang

**Affiliations:** 1 Department of Orthopaedic Surgery, Jeju National University School of Medicine, Jeju, South Korea; 2 Jeju National University Hospital, Aran 13gil 14, Jeju, 63241 South Korea

**Keywords:** Osteoarthritis, Massive rotator cuff tear, Reverse total shoulder replacement

## Abstract

**Background:**

Reverse total shoulder arthroplasty (RTSA) is a treatment option for patients with severe osteoarthritis, rotator cuff arthropathy, or massive rotator cuff tear with pseudoparalysis. We are to deduce not only the early functional outcomes and complications of cementless RTSA during the learning curve period but also complication-based, and operation time-based learning curve of RTSA.

**Methods:**

Between March 2010 and February 2014, we retrospectively evaluated 38 shoulders (6 male, 32 female). The average age of the patients was 73.0 years (range, 63 to 83 years), and the average follow-up was at 24 months (range, 12–53 months). The visual analog scale (VAS), University of California Los Angeles (UCLA) score and constant score were used to evaluate the clinical outcomes. We evaluated patients radiographically at 2 weeks, 3 months, 6 months, 1 year, and then annually thereafter for any evidence of complications.

**Results:**

The VAS score improved from 4.0 to 2.8 (*p* = 0.013). The UCLA score improved from 16.0 to 27.9 (*p* = 0.002), and the constant score improved from 41.4 to 78.9 (*p* < 0.001), which were statistically significant. While active forward flexion, abduction, and internal rotation improved (*p* value = 0.001, < 0.01, 0.15), external rotation did not show significant improvement (*p* = 0.764). Postoperative complications included acromion fracture (one case), glenoid fracture (one case), peripristhetic humeral fracture (one case), axillary nerve injury (one case), infection (one case), and arterial injury (one case). Our study presented an intraoperative complication-based learning curve of 20 shoulders, and operation time-based learning curve of 15 shoulders.

**Conclusions:**

The clinical outcomes of RTSA were satisfactory with overall complication rates of 15.7%. An orthopedic surgeon within the learning curve period for the operation of RTSA should be cautious when selecting the patients and performing RTSA.

**Trial registration:**

Retrospectively registered.

## Introduction

Reverse total shoulder arthroplasty (RTSA) was introduced first by Grammont et al. in 1987 as a treatment for patients with cuff tear arthropathy. Other indications include revision of a failed arthroplasty, malunions of proximal humeral fractures, and pseudoparalysis of the shoulder [1, 2]. The advantage of the design of RTSA was based on the concept of reversing the shoulder joint by fixing a metal ball to the glenoid and introducing a spherical socket into the proximal part of the humerus [3, 4]. This approach lowers the humerus and medializes the center of rotation of the shoulder joint, which increases the deltoid muscle moment arm, allowing for recruitment of more deltoid muscle fibers for arm flexion and abduction [5].

In Europe, RTSA has been performed for more than 20 years. Favard et al. have reported the satisfactory results of long-term follow-ups longer than 10 years [6, 7]. However, it was not approved for use in the USA until 2004, due to highly reported complication rates ranging from 0 to 68% [8]. The most frequent complication is scapular notching followed by complications with the humeral or glenoid component (e.g., loosening) [5]. The rate of humeral loosening is considered to be high for RTSA compared with conventional total shoulder arthroplasty [9]. To avoid the risk of loosening, many surgeons have used cemented components for humeral fixation in RTSA. On the other hand, Michael et al. reported cementless fixation of a porous-coated RTSA humeral stem clinical and radiographic outcomes equivalent to those of cemented stems at minimum 2-years follow-up and mentioned several advantages of cementless fixation: (1) no risk of cement-related complications, (2) decreased operative time, (3) simplified operative technique, and (4) greater ease of revision [10]. Currently, there are convertible modular system RTSA available, which makes easier revision between total shoulder arthroplasty, and RTSA with decrease of surgical time, no removal of well-fixed humeral stem, and excellent post-conversion functional outcomes [11]. The purpose of this study was to analyze the results and complications during the learning curve of cementless RTSA and describe complication based and operation time based learning curve for RTSA.

## Materials and methods

We retrospectively reviewed the charts of 38 consecutive patients who underwent a reverse total shoulder arthroplasty performed by single surgeon between March 2010 and February 2014 and who underwent at least 12 months follow-up. The choice of implant was Comprehensive® reverse shoulder system (Biomet Inc., Warsaw, IN, USA) with cementless cobalt chrome humeral component. All surgical procedures were performed by single orthopedic shoulder surgeon.

The indications for reverse total shoulder arthroplasty were the following: rotator cuff tear arthropathy, massive irreparable rotator cuff tear with chronic loss of elevation that failed to respond to physical treatment, posttraumatic glenohumeral arthritis, and primary osteoarthritis of the shoulder with a massive irreparable cuff tear (Table 1) [12]. Exclusion criteria were poor deltoid function on preoperative electromyography (EMG), a C-spine problem of a related origin, or patients who failed to follow-up.

**Table 1:** Number of cases according to etiology for reverse total shoulder arthroplasty

Indication	Total number of shoulders (*N* = 38)
Rotator cuff tear arthropathy	30 (78.9%)
Irreparable massive cuff tear	5 (13.1%)
Osteoarthritis	3 (7.89%)

Six males and 32 females were enrolled into following research. Twenty-six prostheses were placed in the right shoulder, and 12 were placed in the left shoulder. The average age of the patients was 73 years (range, 63 to 83), with an average follow-up of 24 months (range, 12 to 53 months). On preoperative MRI scan, rotator cuff tears were revealed as follows: 3 cases of 1 tendon tear (8%), 10 cases of 2 tendon tear (26.3%), and 25 cases of 3 tendon tear (65.7%). The IBM SPSS (IBM Co., Armonk, NY, USA) was used for all data analyses. Paired *t* test has been used to compare the preoperative and postoperative clinical scores and range of motion.

All procedures performed in studies involving human participants were in accordance with the ethical standards of the institutional and/or national research committee and with the 1964 Helsinki declaration and its later amendments or comparable ethical standards. As the following study was performed in retrospective manner, formal consent was not required.

### Clinical and radiographic evaluation

All patients were examined preoperatively and postoperatively by two different peer orthopedic surgeons. The visual analog scale (VAS), University of California Los Angeles (UCLA) score, and constant score were used to evaluate the clinical outcomes. Clinically, the range of motion (ROM) of shoulder was measured preoperatively and postoperatively to evaluate the functional outcomes. Patients were asked to perform the following motions: (1) forward flexion, lifting the arm in front of the body, with the palm facing the side of the body and the arm held straight; (2) abduction, arm swinging out from the side of the body, palm facing the side of the body and the arm held straight; (3) external rotation, elbow bent to 90° and swinging the forearm away from the body; and (4) internal rotation, elbow bent to 90° and swinging the forearm toward from the body. We defined the learning curve as the point in the series where there was the lowest risk of complications or leveling out of operative time in subsequent shoulders compared to earlier shoulders.

We evaluated patients radiographically at 2 weeks, 3 months, 6 months, 1 year, and then annually thereafter for any evidence of complications, including changes in the humeral glenoid component position, osteolysis, or scapular notching.

### Operative technique

The surgery was performed with patients in the beach chair position. The deltopectoral anterior approach was used in all cases, and when possible the cephalic vein was protected. The upper portion of the pectoralis major tendon was released, and the medial border of the deltoid muscle was retracted laterally and partially released from its distal insertion by subperiosteal dissection. A longitudinal incision was made through the tendinous portion of the subscapularis muscle and capsule. The subscapularis tendon was tagged with nonabsorbable sutures for easy identification during closure. To expose the humeral head, the humerus was externally rotated and extended. Using a trocar pointed reamer and ratcheting T-handle, a pilot hole was bored through the humeral head along the axis of the humeral shaft, just lateral to the articular surface of humeral head and just posterior to the bicipital groove. The tapered humeral reamer was inserted up to the engraved line above the cutting teeth. The resection guide boom was placed onto the reamer shaft. The prosthesis was implanted at approximately 20° of retroversion. A saw blade was placed through the cutting slot in the guide, and the humeral head was resected. The calcar planer was used to refine the resected surface. A 3.2-mm Steinmann pin was inserted into the glenoid at the desired angle and position. The cannulated baseplate reamer was positioned over the top of the Steinmann pin. The glenoid was reamed to the desired level. After seating the glenoid baseplate, appropriate peripheral screws were inserted. Then select the appropriate glenosphere trial and assemble to a trial taper adaptor. And the assembly was removed from the glenoid baseplate. The glenosphere implant was placed into the impactor base using the glenosphere forceps. After the humeral stem was assembled on to the humeral stem inserter, the stem was inserted into the humeral canal. Appropriate humeral tray and bearing was assembled.

### Postoperative rehabilitation

An abduction brace was applied immediately after surgery and worn for 4 to 6 weeks, and pendulum and early passive wrist and elbow range of motion exercises were initiated at postoperative day 2. Shoulder passive motion exercises were started 2 weeks postoperatively via continuous passive motion machine (ARTROMOT-K1, Ormed GmbH & Co, KG, Germany). After 4 to 6 weeks, the abduction brace was removed, and activity was allowed as tolerated.

## Results

### Functional and clinical outcomes

The average VAS score improved from 4.0 points before surgery to 2.8 points (*p* = 0.013) at the time of follow-up. The average UCLA score improved from 16.0 to 27.9 (*p* = 0.002), and the constant score improved from 41.4 to 78.9 (*p* < 0.001); these increases were statistically significant. Mean forward flexion, abduction, and internal rotation was improved from 99.9°, 69.2°, L5 to 135.4°, 124.8°, L3 respectively. (*p* value = 0.001, < 0.001, = 0.015) However, there was no statistical improvement in external rotation postoperatively (*p* value 0.764) (Table 2).

**Table 2:** Comparison of preoperative and postoperative shoulder functions among 38 patients

	Preoperative	Postoperative	*P* value
UCLA score	16.0 (range 3–35)	27.9 (range 6–35)	0.002*
Constant score	41.4 (range 9–93)	78.9 (range 17–96)	< 0.001*
VAS score	4.0 (range 2–10)	2.8 (range 1–6)	0.013*
ROM (FF)	99.9 (range 25–160)	135.4 (range 30–170)	0.001*
ROM (abd)	69.2 (range 23–91)	124.8 (range 112~140)	< 0.001*
ROM (IR) 90 abd.	L5	L3	0.015*
ROM (ER) 90 abd.	32.4 (range 5–35)	34.0 (range 10–45)	0.764

*Statistically significant

### Radiologic outcomes and complications

**Fig. 1:**
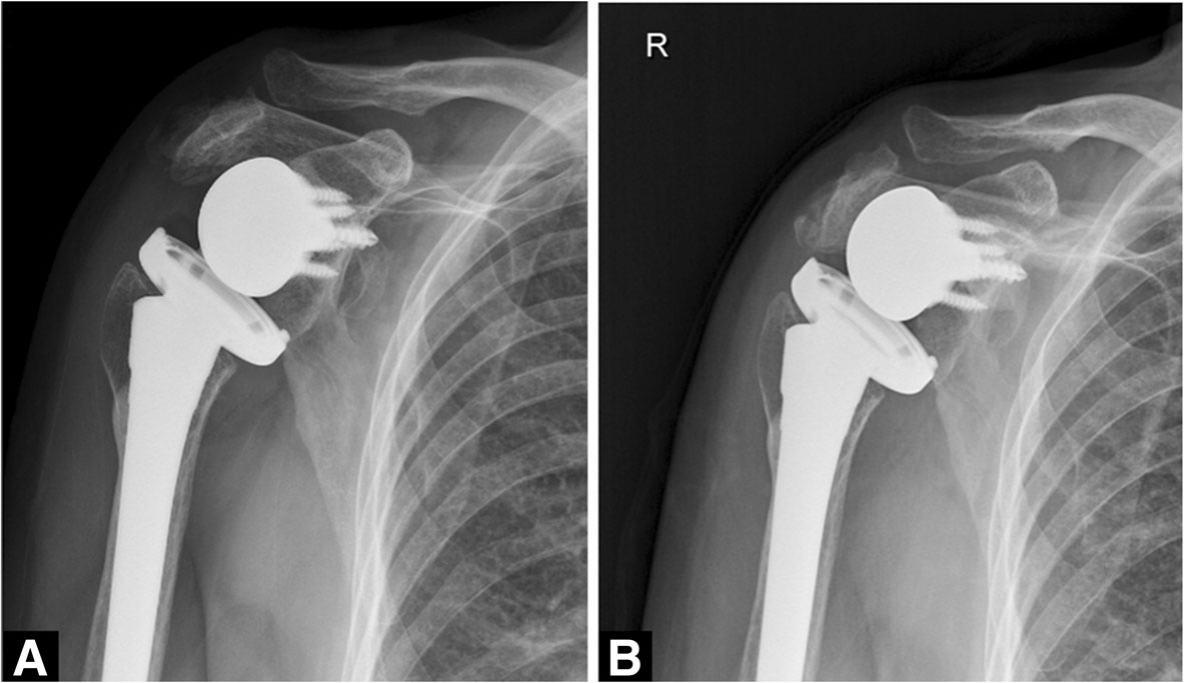
A 79-year-old female patient with acromial fracture. **a** Postoperative anteroposterior radiograph of the shoulder at 18 months follow-up. **b** Anteroposterior radiograph at 22 months follow-up after conservative treatment shows union of the acromion

**Fig. 2:**
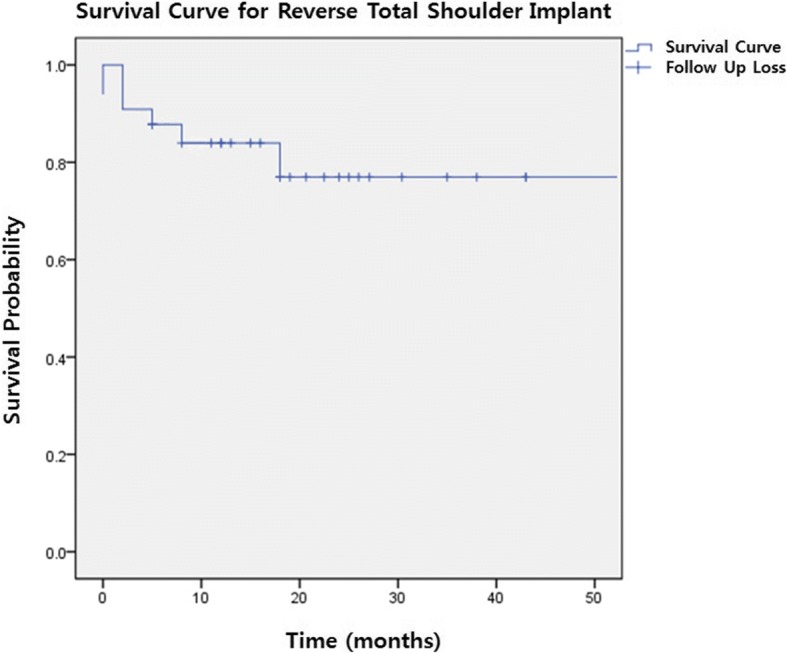
Kaplan-Meier’s survival analysis for reverse total shoulder implant. Following graph shows survival curve for RTSA implant. Survival curve is indicated with solid line. While each descent of curve implies complication, cross symbol (+) implies follow up loss of patient. The survivalship of RTSA implant was revealed to be approximately 76% throughout the follow-up period

The 38 patients were followed for 12 to 53 (mean, 24) months and 6 complications occurred (Table 3). Three patients had fracture in the postoperative period after slipping down; two of the patients were treated conservatively (Fig. 1), and one of them required revision. One patient with superficial infection was resolved with use of IV antibiotics without implant removal. One patient had an injury of the axillary artery intraoperatively and underwent an immediate arterial repair. One patient with axillary nerve palsy resolved itself spontaneously over time without surgical intervention. In our study, all the complications occurred within 2 years after RTSA (Table 3). On radiographic evaluation, there was no evidence of humeral component loosening, osteolysis, or scapular notching. According to Kaplan-Meier’s survival analysis, the survivalship of RTSA implant was revealed to be approximately 76% throughout the follow up period (Fig. 2).

**Table 3:** Intraoperative and postoperative complications after RTSA

Complications	Time from surgery	Treatment and outcomes
Arterial injury	Intraoperative	Arterial repair
Axillary nerve palsy	Postoperative	Complete recovery after 15 months
Periprosthetic humeral fracture	2 months	Revision
Acromion fracture	18 months	Nonoperative
Glenoid fracture	5 months	Nonoperative
Superficial infection	8 months	Nonoperative

### Learning curve

**Fig. 3:**
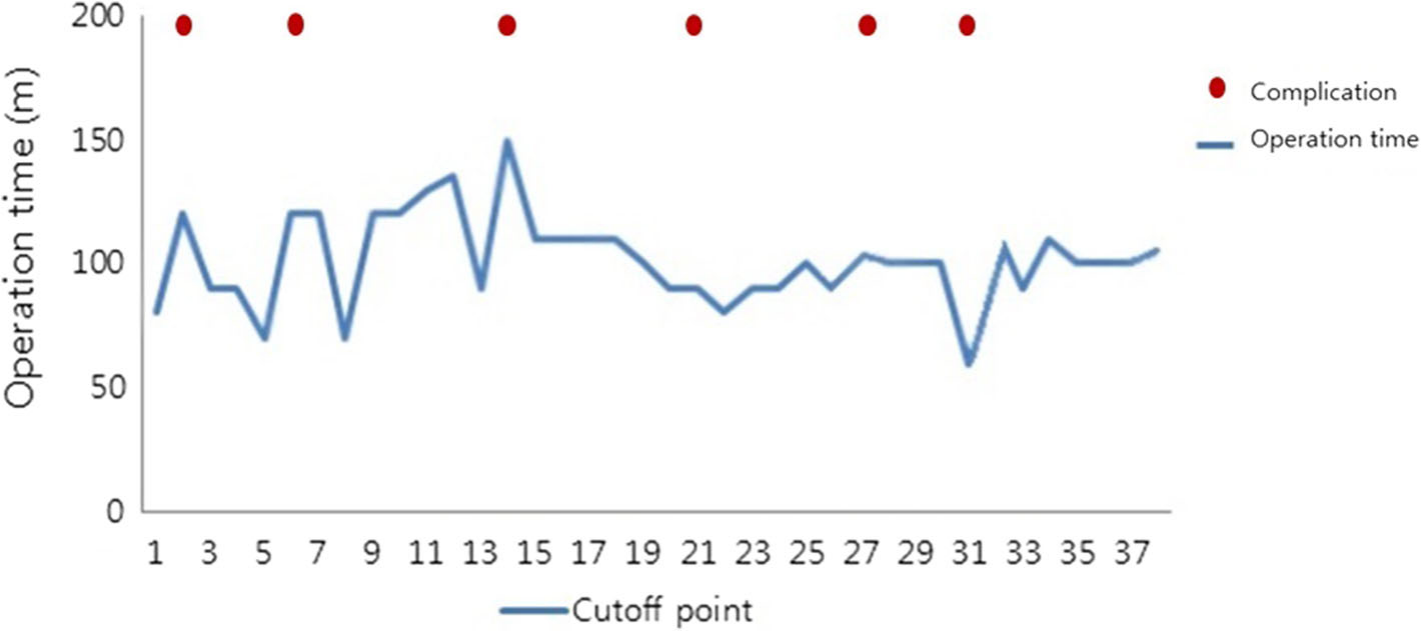
Operation time for each reverse total shoulder arthroplasty. Following graph shows the operation time for consecutive 38 cases of reverse total shoulder arthroplasty. The cutoff points were shown at every three shoulders and significant stabilized and decreased operation time was noted after 15th RTSA. As for cases with intra op, or post complication, there were no discernible pattern

Throughout the consecutive 38 cases of reverse total shoulder arthroplasty, the cutoff points were shown at every 3 shoulders. Complication rate was revealed as 15.7% (6 of 38 patients). Only 2 out of the 6 complications occurred intraoperatively in the first 20 shoulders and 4 occurred after at least 2 months postoperatively. In comparison of operation time between former 18 cases and latter 18 cases, which revealed to be average 108.6 min (range 71~147 min), and average 87.6 min (range 61~121) respectively. It is implied that after gaining certain amount of experience, the decrease of operation time was achieved. Significant decrease of operation time was noted after 15th RTSA (Fig. 3).

## Discussion

The introduction of RTSA represents a new era in shoulder surgery [13]. It could be a treatment option for patients with cuff tear arthropathy or for patients who failed conventional total shoulder arthroplasty. Multiple studies have reported highly variable complication rates of RTSA ranging from 14 to 75% [14]. When selecting patients and deciding to perform RTSA, it is important to consider the complication rates. We therefore described the types and rates of early complications in cementless RTSA during the learning period, characterized a learning curve for our RTSA series to establish where the greatest reduction in operation time and complication rates occurred, and evaluated the clinical and functional outcomes of RTSA.

Some authors reported the short-term clinical and functional outcomes of RTSA appeared to be promising [15]. Sirveaux et al. reported an increase in the mean constant score from 22.6 points preoperatively to 65.6 points postoperatively, with 96% of the patients having little or no pain and an increase in mean active forward flexion from 73° to 138° [16]. Bryan et al. reported that patients who were managed with a RTSA to treat posttraumatic arthritis or a revision arthroplasty had less improvement and higher complication rates than patients with a cuff tear arthropathy or primary osteoarthritis associated with a massive cuff tear [12]. In our study, the VAS, UCLA, constant scores, and range of motion except external rotation were all improved. Since the reverse total shoulder arthroplasty system relies on the deltoid muscle to power and position the arm, instead of the rotator cuff, we do not only think rotator cuff condition is a factor that might have influence over postoperative ROM, but also should not be included as covariate.

The most frequently reported complication of RTSA is scapula notching followed by glenoid and humeral loosening, periprosthetic fracture, acromial fracture, neurological injury, and infection [5]. Several studies reported the variable rates of scapular notching ranging from 0 to 97% [8, 14]. According to Mollon et al. [17], patients with scapular notching present pooper clinical outcomes, less strength, less range of motion, and significantly higher complication rates. Also it is revealed by Roche et al. [18] that scapular notching plays a role in initial glenoid baseplate instability. Considering the consequence of scapular notching, the effort to prevent one cannot be overstated, and every shoulder surgeon who performs reverse total shoulder should be cautious and meticulous when preparing glenoid and placing the baseplate. There were no complications related to humeral component loosening or scapular notching in our study. Large series with long-term follow-up are necessary to properly evaluate scapular notching. However, several studies reported the safest methods to prevent scapular notching are inferior positioning of the glenoid baseplate and larger size implants with shallow concave components [19, 20]. Zumstein et al. reported a combined incidence of acromial and scapular spine fracture of 1.5% (12 out of 782) [21]. Postoperatively, increased deltoid tension and medialization of the center of rotation could increase the load across the acromion. Gerber et al. and Walch reported that 4 of their 58 patients and 4 of 457 patients experienced a postoperative-RSA acromial fracture, respectively [20, 22]. Most acromial fractures can be treated conservatively; however, if the scapular fracture is accompanying an acromial fracture then surgical treatment may be required [4]. The overall incidence of postoperative acromial fracture in our series was 3.0%. They were treated nonoperatively with satisfactory outcomes. As the experience of RTSA gained up, we have adjusted the cutting level of humerus head in order to reduce muscular tension around the implant, which may cause stress fracture of acromion. After placement of trial, check of tension on conjoin tendon of shoulder is made. If the tension is too much, additional 1~2 mm cut of humeral head is performed.

The incidence of infection after RTSA is reported to be 0 to 4%. The prevalence of neurologic injury after RTSA is approximately 1 to 4.3%. A commonly injured nerve is the axillary nerve, which could be injured from direct damage during the surgery, stretch injury from retractors, or postoperative compression of hematoma. In most neurological cases, surgical intervention is not necessary [13, 23]. In our study, there was one patient with axillary nerve palsy resolved spontaneously over time without surgical intervention.

Gilot et al. reported the incidence of radiographic aseptic loosening of the humeral component in RTSA when comparing the cemented and press-fit used group. No loosening occurred in the press-fit group. No statistically significant difference was found in humeral stem loosening [24]. Wiater et al. reported clinical and radiographic results of cementless RTSA. They concluded that there was no significant difference clinically or radiographically between the cemented and cementless groups. They mentioned several advantages of cementless fixation, including no risk of cement-related complications, decreased operative time, simplified operative technique, and greater ease of revision [10]. Bogle et al. reported that cementless trabecular metal porous-coated implants of RTSA are associated with secure glenoid fixation and minimal radiographic evidence of humeral stem loosening or subsidence at short-term follow up [25]. Additionally, trabecular metal (TM) porous-coated ingrowth implants have shown good results and reliability in the total hip arthroplasty and have the potential to provide stable long-term fixation in the shoulder [10, 26]. Because of these advantages, the cementless RTSA could be a good option for a surgeon who is just getting used to the operation.

Sershon et al. reported a 14% complication rate, including 3 revisions within 4 years, after reverse shoulder replacement of 36 shoulders; there was a total survival rate of 91% in patients with a mean age of 54 years [27]. Sirveaux et al. reported that survivorship of the prosthesis was 88% (84 to 92) at 5 years, 71.9% (63 to 81) at 7 years, and 28.8% (7 to 50) at 8 years postoperatively [16].

Previous studies have shown higher complications rates and length of hospital stay for shoulder arthroplasties performed by less experienced surgeons [28, 29]. Rockwood et al. emphasized that only an experienced shoulder surgeon can successfully perform the procedure and is aware of alternative procedures such as the use of hemiarthroplasty [30]. Numerous studies suggest surgeon experience can affect pre- or postoperative clinical results [1, 28, 31]. Wierks et al. reported the learning curve for experienced shoulder surgeon appeared to be seven patients, after which the complication rate decreased. They reported that there were more complications in the first ten procedures performed than in the second ten procedures [8]. According to currently published literatures [8, 14, 32], learning curve was only described by comparison of the complication rate of previously and lately conducted reverse total shoulder arthroplasty, and figure out the point of decrease of complication rate. In our article, we have analyzed 38 cases of reverse total shoulder arthroplasty and tried to define not only the complication-based learning curve but also operation time-based learning curve as well. As for the complication-based learning curve, we have figured out the point of decrease of complication rate throughout the series of 38 cases. The whole complication rate revealed to be 15.7% (6 of 38 patients). Only 2 out of the 6 complications occurred intraoperatively in the first 20 shoulders and 4 occurred after at least 2 months postoperatively. However, there were no discernible patterns among intraoperative and postoperative complications. As for operation time-based learning curve, significant stabilized and decreased operation time was noted after 15th RTSA. In summary, intraoperative complication based-learning curve and operation time based-learning curve verified by our study is 20 cases and 15 cases respectively.

The following study has several limitations. First, the sample size was small. Second, the follow-up period is quite short (24 months) and the complication rate might increase with time. Previous studies with long-term follow-up showed increased complication rates. Third, the study was conducted only using Comprehensive® reverse shoulder system (Biomet Inc., Warsaw, IN, USA) with cementless humeral component, and it has not only been compared to its contemporaries and their survivorship but also to the cemented RTSA systems. Lastly, we have not compared the clinical outcomes and complications of RTSA between ones that performed during learning curve phase and expert phase.

## Conclusion

The short-term follow-up of cementless RTSA showed satisfactory early clinical and functional outcomes; however, given the relatively high complication rate, further study with long-term follow-up is required. The orthopedic surgeon must be cautious when deciding to perform RTSA unless he or she is familiar with the anatomy and function of the shoulder. Acquired experience will help surgeons refine patient selection with greater confidence in the procedure and decease the operation time, yielding more satisfactory and promising clinical outcomes.

## Abbreviation

RTSA: Reverser total shoulder arthroplasty

## References

[1] Werner C, Steinmann P, Gilbart M, Gerber C (2005). Treatment of painful pseudoparesis due to irreparable rotator cuff dysfunction with the Delta III reverse-ball-and-socket total shoulder prosthesis. JBJS.

[2] Hovorka I (2006). The Grammont reverse shoulder prosthesis: results in cuff tear arthritis, fracture sequelae, and revision arthroplasty. J Shoulder Elbow Surg.

[3] Grammont PM, Baulot E (2011). The classic: delta shoulder prosthesis for rotator cuff rupture. Clin Orthop Relat Res.

[4] Cheung E, Willis M, Walker M, Clark R, Frankle MA (2011). Complications in reverse total shoulder arthroplasty. JAAOS-J Am Acad Orthop Surg.

[5] Farshad M, Gerber C (2010). Reverse total shoulder arthroplasty—from the most to the least common complication. Int Orthop.

[6] Favard L, Levigne C, Nerot C, Gerber C, De Wilde L, Mole D (2011). Reverse prostheses in arthropathies with cuff tear: are survivorship and function maintained over time?. Clin Orthop Relat Res.

[7] Guery J, Favard L, Sirveaux F, Oudet D, Mole D, Walch G (2006). Reverse total shoulder arthroplasty: survivorship analysis of eighty replacements followed for five to ten years. JBJS.

[8] Wierks C, Skolasky RL, Ji JH, McFarland EG (2009). Reverse total shoulder replacement: intraoperative and early postoperative complications. Clin Orthop Relat Res.

[9] Favard L, Katz D, Colmar M, Benkalfate T, Thomazeau H, Emily S (2012). Total shoulder arthroplasty–arthroplasty for glenohumeral arthropathies: results and complications after a minimum follow-up of 8 years according to the type of arthroplasty and etiology. Orthop Traumatol Surg Res.

[10] Wiater JM, Moravek JE, Budge MD, Koueiter DM, Marcantonio D, Wiater BP (2014). Clinical and radiographic results of cementless reverse total shoulder arthroplasty: a comparative study with 2 to 5 years of follow-up. J Shoulder Elb Surg.

[11] Williams PN, Trehan SK, Tsouris N, Dines JS, Dines DM, Craig EV, Gulotta LV, Warren RF (2017). Functional outcomes of modular conversion of hemiarthroplasty or total to reverse total shoulder arthroplasty. HSS J.

[12] Wall B, O'Connor DP, Edwards TB, Nové-Josserand L, Walch G (2007). Reverse total shoulder arthroplasty: a review of results according to etiology. JBJS.

[13] Laedermann A, Lübbeke A, Melis B, Stern R, Christofilopoulos P, Bacle G, Walch G (2011). Prevalence of neurologic lesions after total shoulder arthroplasty. JBJS.

[14] Kempton LB, Ankerson E, Wiater JM (2011). A complication-based learning curve from 200 reverse shoulder arthroplasties. Clin Orthop Relat Res.

[15] Frankle M, Siegal S, Pupello D, Saleem A, Mighell M, Vasey M (2005). The reverse shoulder prosthesis for glenohumeral arthritis associated with severe rotator cuff deficiency: a minimum two-year follow-up study of sixty patients. JBJS.

[16] Sirveaux F, Favard L, Oudet D, Huquet D, Walch G, Mole D (2004). Grammont inverted total shoulder arthroplasty in the treatment of glenohumeral osteoarthritis with massive rupture of the cuff: results of a multicentre study of 80 shoulders. Bone & Joint J.

[17] Mollon B, Mahure SA, Roche CP, Zuckerman JD (2017). Impact of scapular notching on clinical outcomes after reverse total shoulder arthroplasty: an analysis of 476 shoulders. J Shoulder Elb Surg.

[18] Roche CP, Stroud NJ, Martin BL, Steiler CA, Flurin P-H, Wright TW, DiPaola MJ, Zuckerman JD (2013). The impact of scapular notching on reverse shoulder glenoid fixation. J Shoulder Elb Surg.

[19] Gutiérrez S, Luo Z-P, Levy J, Frankle MA (2009). Arc of motion and socket depth in reverse shoulder implants. Clin Biomech.

[20] Gerber C, Pennington SD, Nyffeler RW (2009). Reverse total shoulder arthroplasty. JAAOS-J Am Acad Orthop Surg.

[21] Zumstein MA, Pinedo M, Old J, Boileau P (2011). Problems, complications, reoperations, and revisions in reverse total shoulder arthroplasty: a systematic review. J Shoulder Elb Surg.

[22] Walch G, Mottier F, Wall B, Boileau P, Molé D, Favard L (2009). Acromial insufficiency in reverse shoulder arthroplasties. J Shoulder Elb Surg.

[23] Lynch NM, Cofield RH, Silbert PL, Hermann RC (1996). Neurologic complications after total shoulder arthroplasty. J Shoulder Elb Surg.

[24] Gilot G, Alvarez-Pinzon AM, Wright TW, Flurin P-H, Krill M, Routman HD, Zuckerman JD (2015). The incidence of radiographic aseptic loosening of the humeral component in reverse total shoulder arthroplasty. J Shoulder Elb Surg.

[25] Bogle A, Budge M, Richman A, Miller RJ, Wiater JM, Voloshin I (2013). Radiographic results of fully uncemented trabecular metal reverse shoulder system at 1 and 2 years' follow-up. J Shoulder Elb Surg.

[26] Corten K, Bourne RB, Charron KD, Au K, Rorabeck CH (2011). Comparison of total hip arthroplasty performed with and without cement: a randomized trial. J Bone Joint Surg (Am Vol).

[27] Sershon RA, Van Thiel GS, Lin EC, McGill KC, Cole BJ, Verma NN, Romeo AA, Nicholson GP (2014). Clinical outcomes of reverse total shoulder arthroplasty in patients aged younger than 60 years. J Shoulder Elb Surg.

[28] Hammond JW, Queale WS, Kim TK, McFarland EG (2003). Surgeon experience and clinical and economic outcomes for shoulder arthroplasty. JBJS.

[29] Jain N, Pietrobon R, Hocker S, Guller U, Shankar A, Higgins LD (2004). The relationship between surgeon and hospital volume and outcomes for shoulder arthroplasty. JBJS.

[30] Rockwood CA Jr. The reverse total shoulder prosthesis: the new kid on the block: LWW; 2007. 10.2106/JBJS.F.01394 17272434

[31] Boulahia A, Edwards TB, Walch G, Baratta RV (2002). Early results of a reverse design prosthesis in the treatment of arthritis of the shoulder in elderly patients with a large rotator cuff tear. Orthopedics.

[32] Cho C-H, Song K-S, Koo T-W (2017). Clinical outcomes and complications during the learning curve for reverse total shoulder arthroplasty: an analysis of the first 40 cases. Clinics in orthopedic surgery.

